# Direct Deposition of Gas Phase Generated Aerosol Gold Nanoparticles into Biological Fluids - Corona Formation and Particle Size Shifts

**DOI:** 10.1371/journal.pone.0074702

**Published:** 2013-09-27

**Authors:** Christian R. Svensson, Maria E. Messing, Martin Lundqvist, Alexander Schollin, Knut Deppert, Joakim H. Pagels, Jenny Rissler, Tommy Cedervall

**Affiliations:** 1 Division of Ergonomics and Aerosol Technology, Department of Design Sciences, Lund University, Lund, Sweden; 2 Division of Solid State Physics, Department of Physics, Lund University, Lund, Sweden; 3 Division of Synchrotron Radiation Research, Department of Physics, Lund University, Lund, Sweden; 4 Department of Biochemistry and Structural Biology, Lund University, Lund, Sweden; National Institute of Health (NIH), United States of America

## Abstract

An ongoing discussion whether traditional toxicological methods are sufficient to evaluate the risks associated with nanoparticle inhalation has led to the emergence of Air-Liquid interface toxicology. As a step in this process, this study explores the evolution of particle characteristics as they move from the airborne state into physiological solution. Airborne gold nanoparticles (AuNP) are generated using an evaporation-condensation technique. Spherical and agglomerate AuNPs are deposited into physiological solutions of increasing biological complexity. The AuNP size is characterized in air as mobility diameter and in liquid as hydrodynamic diameter. AuNP:Protein aggregation in physiological solutions is determined using dynamic light scattering, particle tracking analysis, and UV absorption spectroscopy. AuNPs deposited into homocysteine buffer form large gold-aggregates. Spherical AuNPs deposited in solutions of albumin were trapped at the Air-Liquid interface but was readily suspended in the solutions with a size close to that of the airborne particles, indicating that AuNP:Protein complex formation is promoted. Deposition into serum and lung fluid resulted in larger complexes, reflecting the formation of a more complex protein corona. UV absorption spectroscopy indicated no further aggregation of the AuNPs after deposition in solution. The corona of the deposited AuNPs shows differences compared to AuNPs generated in suspension. Deposition of AuNPs from the aerosol phase into biological fluids offers a method to study the protein corona formed, upon inhalation and deposition in the lungs in a more realistic way compared to particle liquid suspensions. This is important since the protein corona together with key particle properties (e.g. size, shape and surface reactivity) to a large extent may determine the nanoparticle effects and possible translocation to other organs.

## Introduction

As the use of manufactured nanoparticles (MNPs) increase, so does the risk of exposure. There is a growing concern that nanoparticles may pose a hazard to human health. Some MNPs commonly used today are carbon based, or formed by metal and metal oxides such as gold, silver, zinc [1]–[3] or titanium dioxide [4]. As the particle sizes decrease towards the nanometer scale, materials that are non-toxic in bulk form may become toxic. Thus, the need for nanoparticle toxicological data is greater than ever. The interaction of nanoparticle with biological systems is, however, not trivial to study. Knowledge about how nanoparticles enter the body and how the exposure route alters their possible toxicity could greatly facilitate cost-effective approaches to decrease the exposure of the most harmful particles, and thus ensure a safe future development of nanotechnology. Examples of exposure routes are inhalation, skin and eyes, where inhalation has been defined as being one of the most important exposure routes from a health risk perspective [5]. The deposition probability of inhaled nanoparticles in the lung as a function of particle diameter has been carefully studied for spherical and agglomerated as well as hydrophobic and hydrophilic particles [6], [7].

One type of interaction that is believed to be of relevance for these observations is that between nanoparticles and proteins. Once deposited in the lungs, the nanoparticles encounter a biological environment and are immediately covered with proteins and other biomolecules [8]–[10]. The bimolecular corona formed is hypothesized to be the true entity that determines the biological fate and effects of the nanoparticles [11]. Particles of different characteristics have been shown to generate different coronas in various protein solutions [12], [13]. Toxicological studies have shown that the choice of solute can play a major role in the level of toxicity the particles can exert [14], [15]. This means that particles ultimately could have different biological effects depending on the surrounding environment. The size, morphology and aggregation state of particles have also been shown to be important for the particles’ toxicological responses [16], [17]. The size and aggregation state of particulate matter in aqueous solutions is known to be effected by the corona formed on the particles’ surface. Thus, in order to understand the fate of particles upon deposition in the lung lining fluid and the subsequent biological effects, it is important to understand the formation of the particle protein corona, the aggregation state and hydrodynamic size. This is also crucial when interpreting observations of toxicological tests, both *in vivo* and *in vitro*, when performed using liquid suspensions of nanoparticles and when designing new studies of particle toxicology.

Many experiments have been performed *in vivo* and *in vitro* to investigate the above mentioned properties and their importance for the toxicology of inhalable particles [18], [19]. For such tests to provide relevant results, they need to mimic the real exposure situation as closely as possible. At the least, we need to understand the potential effect introduced by the differences from the real exposure situation. Traditionally, toxicological studies have been performed using particle liquid suspension – both *in vivo* [20]–[22] and *in vitro* (submerged cell-culture studies) [23]–[26]. These methods of experimentation in complete suspension, using functionalized nanoparticles, have been questioned with regards to their physiological relevance for exposure via the inhalation route. Can functionalized nanoparticles administered in completely aqueous cell systems produce reliable toxicological data for inhaled nanoparticles? For example, the coating of the particles might be different and particles in suspension would rely on mechanisms such as sedimentation and Brownian diffusion to come into contact with cells adhered to a surface [27]. This can for example introduce a bias when investigating size and surface area related toxic effects if not accounted for.

An alternative to traditional toxicology is to deposit aerosol particles directly from the gas phase onto the cell surface by using air-liquid interface deposition chambers [23], [27]–[29]. This mimics more closely an inhalation exposure situation. Here we apply this principle to deposit airborne particles directly into solutions of biomolecules to study the corona formation, without having the impact of other surfactant molecules on the nanoparticle. Thus, by generating pure uncoated particles with well-known characteristics in the gas phase followed by immediate deposition, many of the experimental drawbacks of the liquid suspension can be overcome [29], [30].

This study employs a novel combination of techniques to determine how particle properties are manifested from an air-liquid interface perspective. Uncoated gold nanoparticles (AuNP) were generated in the gas phase by an evaporation and condensation technique [31]. They were characterized in the aerosol phase within seconds of their formation using a Differential Mobility Analyzer (DMA) and an Aerosol Particle Mass Analyzer (APM) [32], [33], characterizing the AuNP with respect to size and mass respectively. Further, particles were collected for Transmission Electron Microscopy image analysis (TEM). After charging, the nanoparticles are deposited by electrostatic forces into solutions of increasing complexity: homocysteine, bovine serum albumin (BSA), porcine blood serum, and lung fluid. The lung fluid system is of special interest since one of the primary exposure routes for nanoparticles is by the respiratory system. Particles in the solutions were characterized using dynamic light scattering (DLS), particle tracking analysis (PTA) and UV absorbance spectroscopy. Protein corona analysis was performed on the AuNP in physiological solutions, and the total AuNP surface area was related to the homocysteine/protein surface area in solution. The relevant size properties for diffusional transport were measured both in air (mobility diameter) by the DMA and in liquid solution (hydrodynamic diameter) by DLS and PTA.

## Results and Discussion

### Method Overview

In this study we develop a method for deposition of airborne AuNPs into the liquid phase. We show using a set of characterization methods that the particles are successfully deposited into the liquid, with a varying degree of aggregation and hydrodynamic sizes depending on the solution used.

**Figure 1:**
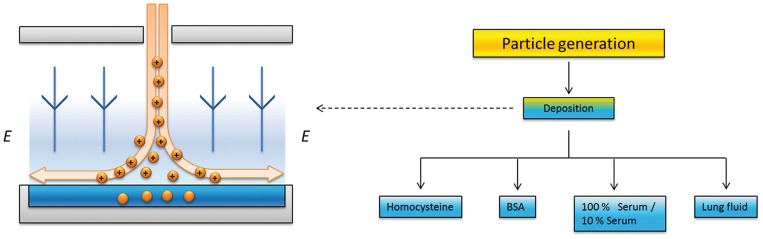
Overview of the generation and deposition strategy for the study. Particles are generated in the aerosol phase and characterized, led to the deposition chamber, and deposited into various physiological solutions of increasing biological complexity, followed by characterization in the solution.

**Figure 2:**
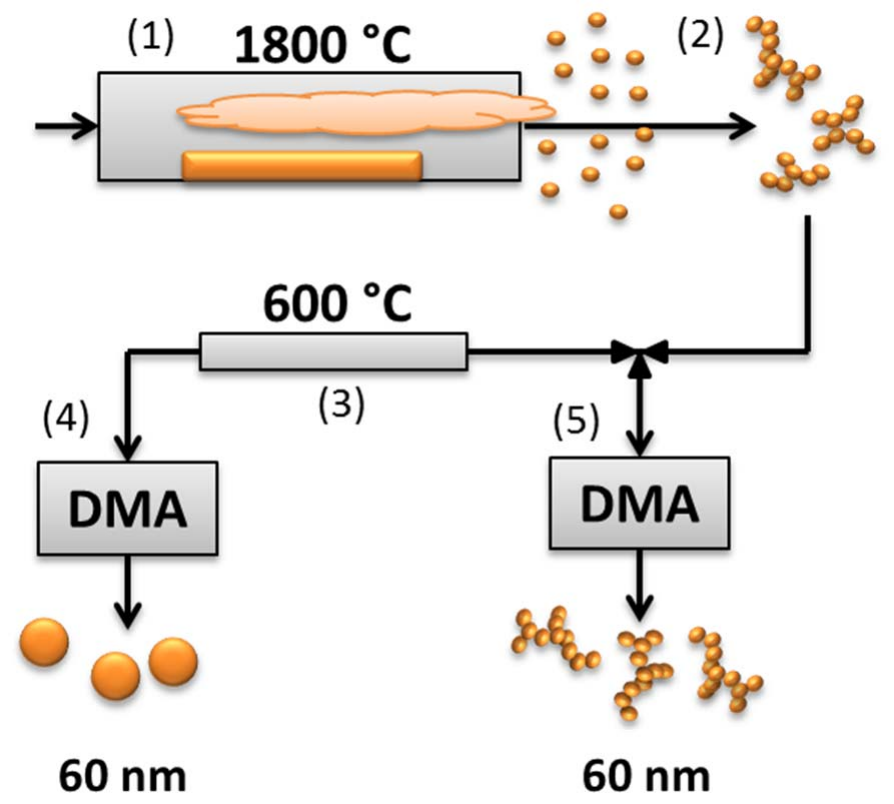
Principle of particle generation, sintering and size selection process. 1) Evaporation condensation furnace, formation of primary particles 5–6 nm. 2) Primary particles coagulate into larger agglomerates. 3) Sintering furnace, allows for generation of spherical AuNP. 4) Spherical 60 nm AuNP are selected from the airborne particle stream using a DMA. 5) Agglomerate 60 nm AuNP are selected from the airborne particle stream using a DMA.

AuNPs are generated and characterized with respect to size, particle mass and shape while airborne. Two types of particles are generated: 1) spherical with a mobility diameter of 60 nm and 2) agglomerates of the same mobility diameter, consisting of smaller primary particles of ∼5–6 nm. Most experiments are performed using spherical particles. The particles are charged and deposited in various physiological fluids by electrostatic precipitation. An overview of the experimental and generation procedure is shown in Figure 1 and 2.

### Particle Generation and Characterization in the Aerosol Phase

Figure 2 shows a conceptual scheme of the generation of agglomerate and sintered AuNPs. The heating of bulk material generates a saturated metal vapor. Upon cooling, the vapor immediately nucleates, followed by condensation into so called primary particles, 5–6 nm in diameter. These in turn aggregate into larger metal agglomerates by coagulation in the gas-phase. To form spherical AuNPs the agglomerates are compacted while airborne, this process is known as sintering. This is done by letting the particles pass through a tube furnace where the particles are exposed to near melting point temperatures, causing the primary particles of an agglomerate to coalesce into essentially spherical shapes. The generated particles – spheres or agglomerates – pass a DMA, selecting only particles of one size. In our study the selected particles correspond to a diameter of 60 nm. Hence either an agglomerate or sintered airborne particle stream of the same mobility diameter can be generated. Thus, specific particle sizes can be selected and deposited into various biological fluids. While airborne the particles are characterized with respect to size, particle mass and shape, followed by deposition using electrostatic precipitation.

**Figure 3:**
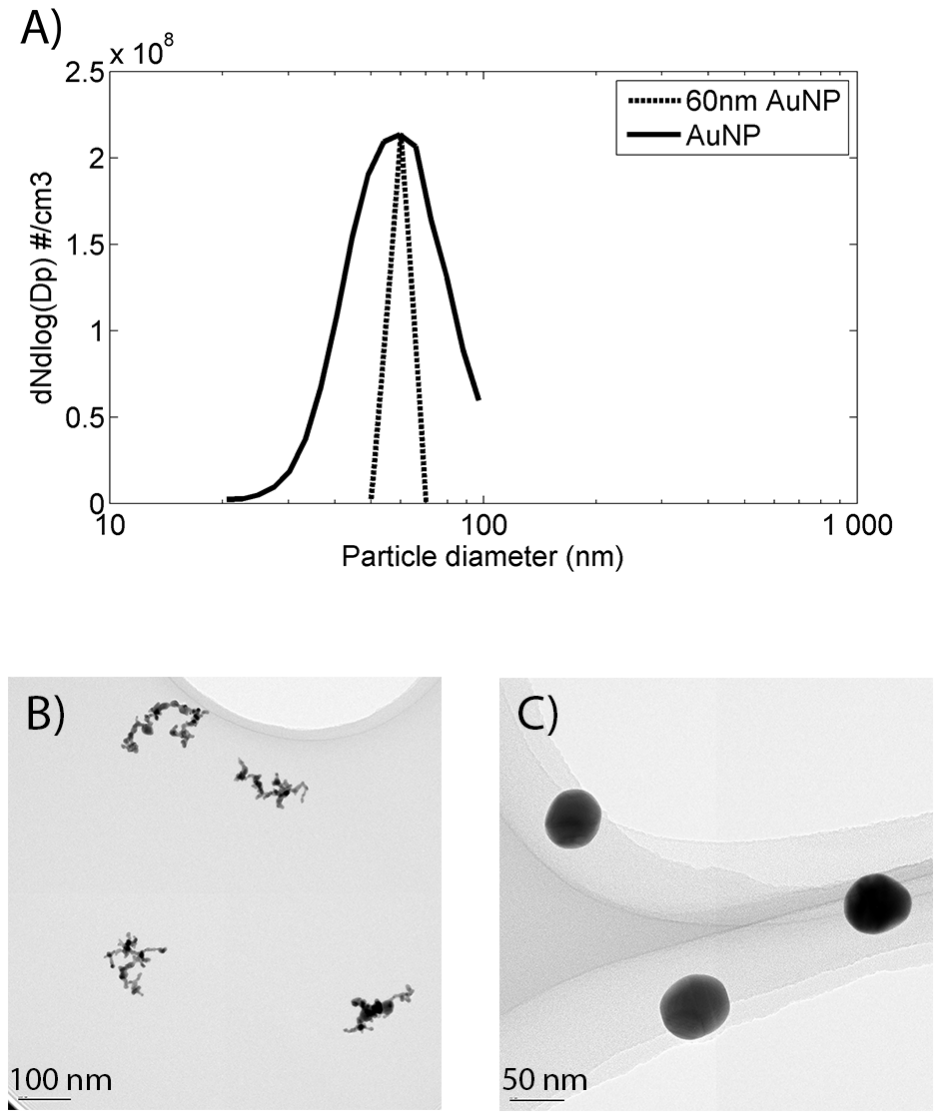
Airborne AuNP characteristics. A) The original number size distribution of the sintered AuNP aerosol, as well as the monodisperse particles for deposition in physiological solution (60 nm). B) 60 nm AuNP agglomerates visualized by TEM. C) 60 nm sintered spherical AuNPs visualized by TEM.

Figure 3A show the particle size distribution (particle number concentration as a function of particle size), of the spherical AuNPs before and after selection by the DMA. The size distribution is characterized using a scanning mobility particle sizer (SMPS), further described in the Materials and Methods section. The means of visualization is common in the field of aerosol technology and is given as *dN/dlogDp*, where *N* is the number concentration of particles and *D_p_* the particle diameter [34]. From the generated distribution the size fraction selected for deposition is visualized (Figure 3A). The width of the distribution of the selected particles is determined by the so called transfer function of the DMA. Figure 3B and C shows the transmission electron microscopy imagery (TEM) of the selected particles deposited on carbon-coated copper grids.

The number concentration of 60 nm agglomerate AuNP is approximately 2 times lower than for the spherical AuNP. This is due to a higher generation temperature in the evaporation- condensation furnace needed to generate particles of high enough mass to get 60 nm particles after sintering. The agglomerates are less dense and less material is needed to generate 60 nm agglomerates. The spherical and agglomerate AuNP mass and particle density is determined by using an aerosol particle mass analyzer (APM) operated downstream the DMA [33], [35], [36]. Spherical AuNPs of 60 nm mobility diameter, with a density of 16,3 g/cm^3^, have a mass of approximately 1.8±0.037 fg/particle. The spherical AuNP density is slightly lower than the ideal bulk density of gold. AuNP agglomerates of 60 nm mobility diameter have a mass of approximately 0.6±0,012 fg/particle.

The probability of a nanoparticle to be deposited in the respiratory tract is governed by the diffusivity of the particles in air when transported in the lung (as Brownian diffusion is the driving force of deposition). Since the mobility diameter can be directly related to the particle diffusivity in air, AuNP spheres and agglomerates, with the same mobility diameter, have a similar probability of deposition in the human. However, the mass is larger for the spherical particles, resulting in higher mass doses even if the deposited number concentration is the same.

For airborne AuNPs, the measured equivalent size is the electrical mobility diameter, an equivalent size determining diffusive transport of nanoparticles in air [37]. For AuNPs in solution, the size measurements are based on the hydrodynamic diameter, with the exception of absorbance. The hydrodynamic diameter is, as the mobility diameter, an equivalent size describing the diffusivity of the particle. For physically spherical particles (as for the sintered AuNPs in this study before deposition), the electrical mobility diameter and hydrodynamic diameter by default equals the true physical diameter. Both these equivalent diameters are independent of particle density and mainly determined by the particle volume and morphology. For agglomerates, consisting of smaller fused primary particles, the size represents an equivalent diameter of a spherical particle that has the same diffusivity as the agglomerate in respective media (air/N_2_ and physiological fluids). From the results we find that the hydrodynamic diameter in liquids is larger than the mobility diameter in air in all cases. This is primarily due to protein adsorption forming a corona on the AuNP.

### Deposition of Spherical AuNP in Physiological Solutions of Increasing Complexity

AuNPs were deposited into a 10 mM NaPO_4_ buffer with 5 mM homocysteine. Homocysteine is a natural occurring non-protein amino acid. The rationale for using homocysteine is that the thiol group will bind to the gold surface covalently and the charged carboxyl and amine groups will interact with water and potentially create repulsion between the particles and thereby inhibit particle aggregation. Spherical 60 nm AuNPs are deposited into homocysteine buffer, resulting in an AuNP concentration of approximately 0.17±0.02 mg/ml. The concentration is estimated assuming a deposition efficiency of 100% [38], see material and methods for further details. The Z-average hydrodynamic particle diameter in the homocysteine AuNP solution is approximately 1300 nm. The results indicate that homocysteine is not able to promote individual AuNP:homocysteine complex formation in solution. Larger gold-flakes (<mm) can be visually observed in the sample.

**Figure 4:**
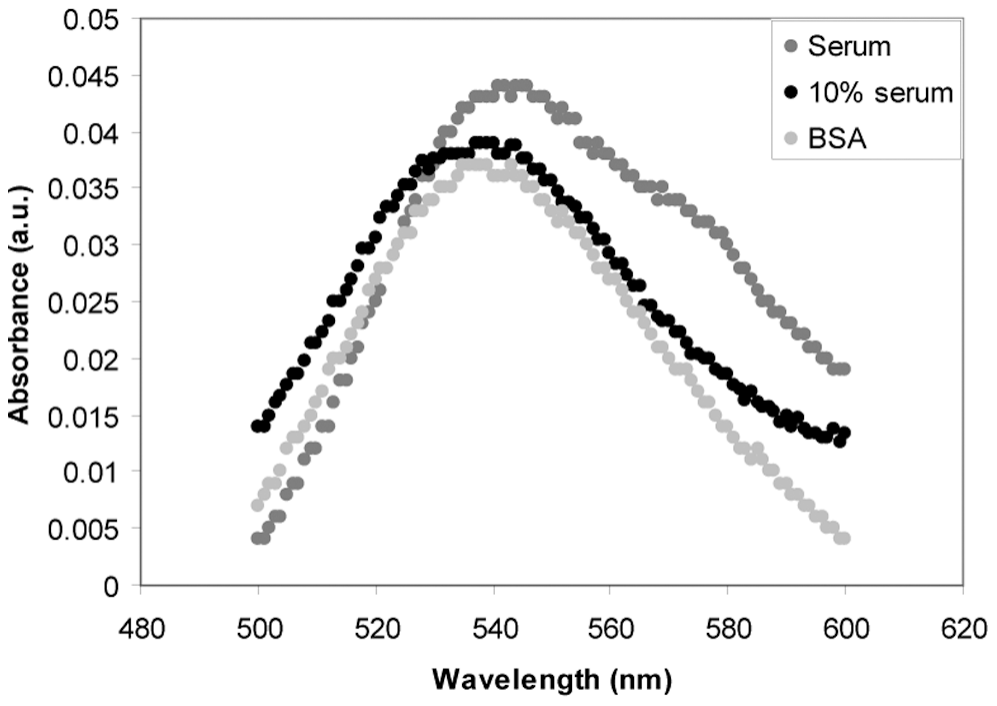
UV absorbance spectra of AuNPs in BSA and porcine blood serum solutions.

Spherical 60 nm AuNPs are also deposited into 35 mg/ml BSA in 10 mM NaPO_4_. Serum albumin is the most abundant protein in blood serum and its concentration in serum is approximately 35 mg/ml. Proteins, among them albumin, are known to bind AuNPs in solution and form discrete complexes [39] and to disaggregate some types of nanoparticles, forming smaller aggregates or individual particles [40]. The hypothesis is that uncoated AuNPs deposited into a BSA solution will associate with albumin, which will hinder aggregation of the particles. After deposition a pink surface layer is observed, not observed when the AuNPs were deposited into the homocysteine solution. The layer dissolves after repeated pipetting of the solution. Albumin and other proteins in solution are known to form a surface to air layer [41]. It is possible that the AuNPs are trapped in the surface layer. This film is observed on top of all biological fluids in the study after deposition of spherical AuNPs, except for homocysteine. Preliminary results indicate that the layer is stable over time (days) and that AuNPs do not diffuse into the surrounding solution. The AuNP concentration in the BSA solution after deposition is calculated to 11.7±0.5 µg/ml, see material and methods for further details. The AuNPs in the resulting solution are characterized by UV absorbance measurements from 500 to 600 nm (Figure 4). The absorbance background for the physiological fluids is deducted prior to the measurements. After subtraction of the absorbance of the BSA solution without AuNPs, there is an absorbance peak at approximately 540 nm. This is the expected wavelength peak from the plasmon absorbance from spherical gold particles with a diameter of 60 nm [42], which corresponds to the size of the deposited spherical AuNP, suggesting that the gold particles are monodisperse in solution. This, together with the observation of the pink surface layer, suggests that the deposited AuNPs have formed AuNP:Protein complexes rather than AuNP:AuNP complexes in solution.

**Figure 5:**
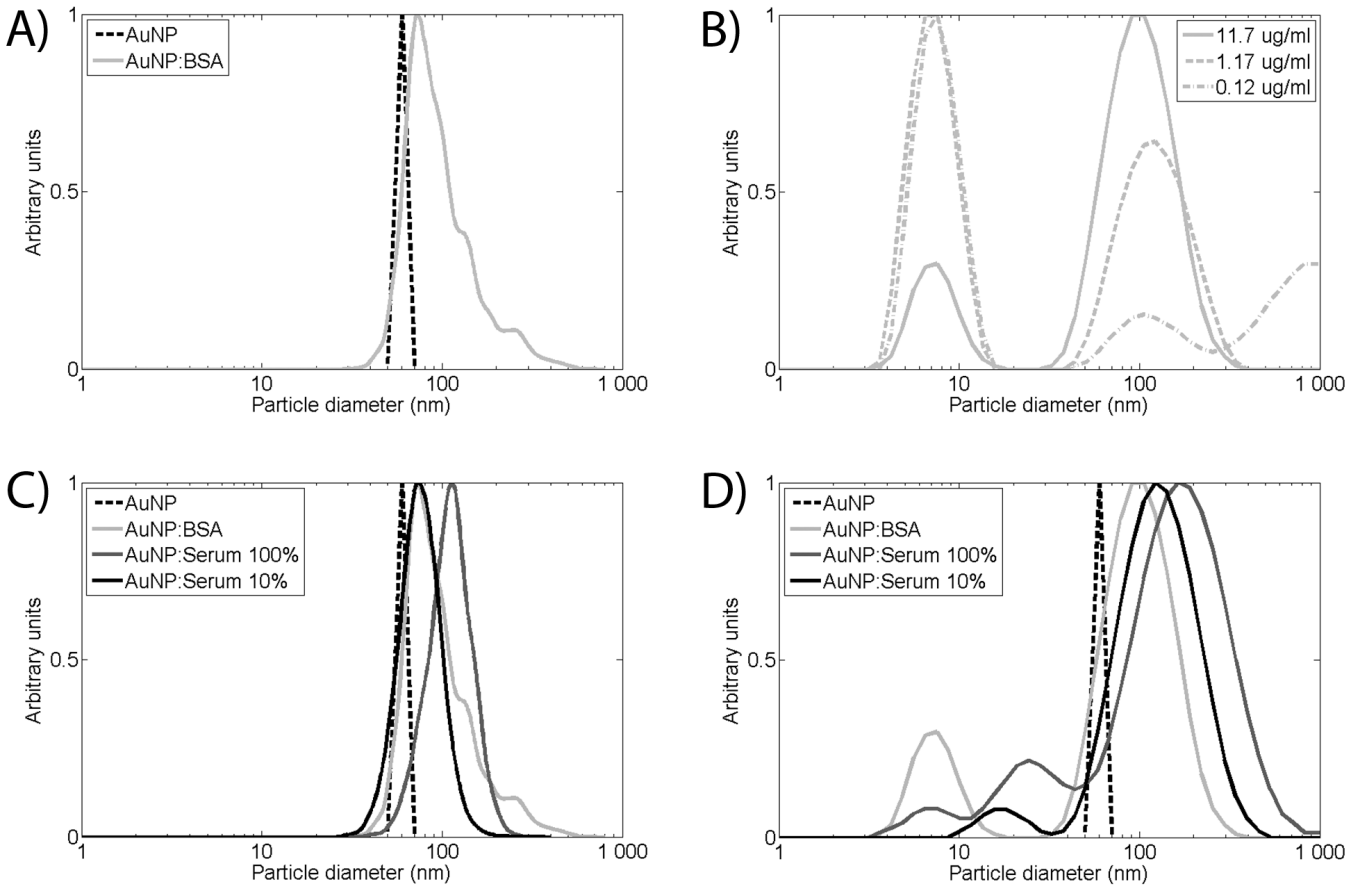
Size distributions of AuNPs after deposition into biological fluids, dashed line represents AuNP aerosol fraction selected for deposition. A) PTA measurement of AuNP in BSA solution. B) DLS measurements of AuNP in BSA solution at three different dilution levels in BSA buffer. C) PTA measurements for AuNP in 100% serum, 10% serum and BSA buffer. D) DLS measurements for AuNPs in 100% serum, 10% serum and BSA. The DLS distributions are intensity weighted in the figure.

The size of AuNP:BSA complexes are measured by PTA (Figure 5A and Table 1). Before the measurements, the AuNP sample is diluted 100 times with MQ water. This is done to reduce sample viscosity. The AuNP:BSA complexes show a peak corresponding to 80±3 nm in hydrodynamic diameter. Experiments show that albumin can be approximated by a rectangular structure of approximately 8.0 nm · 7.3 nm, from a cross sectional perspective [43]. Consequently, an albumin monolayer on the AuNP surface will increase the diameter by approximately 14.6–16.0 nm., depending on the protein conformation on the AuNP surface Adding these values to the size of deposited AuNPs, also taking ±10% on the AuNP particle size into account, the theoretical diameter of a AuNP:BSA complex size can be estimated in solution to 69–81 nm, assuming an average of the two conformational states of albumin. The measured complex diameter, measured by PTA, fell well within the range of the theoretically expected hydrodynamic diameter.

**Table 1:** Hydrodynamic sizes of the AuNP:Protein complexes by DLS and PTA along with the standard deviation of the mean value estimated from repeated measurements.

	AuNP (µg/ml ± std)	DLS diameter (nm ± std)	PTA diameter (nm ± std)
BSA	11.7±0.5	109±7/7.6±0.4	80±3^1^
BSA	1.17	7.3±0.1/145±22	–
BSA	0.117	7,7±0.1/156±112	–
100% Serum	16.7±2,5	198±12/25±8	113±6^2^
10% Serum	19.2±2,9	142±8/14±7	75±1^3^
Lung Fluid	16.7±2,5	265±17/22±12	110±7^4^

The peaks representing AuNP:Protein complexes are denoted by bold text. Where two sizes are presented, the first one is the dominating. The diameter refers to the peak mode diameter for both DLS and PTA. In the experiment spherical AuNPs were used.

1 The actual AuNP concentration during PTA measurements was 11.7·10^−2^ µg/ml due to dilution.

2 The actual AuNP concentration during PTA measurements was 16.7·10^−5^ µg/ml due to dilution.

3 The actual AuNP concentration during PTA measurements was 19.2·10^−4^ µg/ml due to dilution.

4 The actual AuNP concentration during PTA measurements was 16.7·10^−2^ µg/ml due to dilution.

In addition to PTA, the size of the AuNPs is analyzed by DLS (Figure 5B). The solution of AuNP:BSA complexes is diluted with pure BSA in PBS solution to 1.17 and 0.117 µg/ml, effectively lowering the ration AuNP to BSA over two orders of magnitude. This was performed to detect any concentration dependency in the measured size of the AuNP:BSA complex. The size intensity signature from all dilutions shows, by distribution analysis, two distinct peaks (Figure 5B). One peak represents a size of 7–8 nm, which corresponds well with the expected hydrodynamic diameter of BSA. The other peak represents an AuNP:BSA complex. The ratio between BSA and the AuNP:BSA complex change when the AuNPs are diluted with the BSA buffer as it would be expected. For the highest concentration, the AuNP:BSA complex peak dominates; for the intermediate concentration the peaks are similar; and for the lowest concentration of the AuNP:BSA complex, the BSA peak dominates. The average sizes of the AuNP:BSA complex peaks are 109±7, 145±22, and 156±112 nm hydrodynamic diameter for 100%, 10% and 1% AuNP concentrations, respectively (Table 1). The high standard deviation for the 1% case is primarily due to one measurement, if removed the standard deviation drop to 18 nm.

An increase in particle size with increasing dilution of particles is expected. This is because in high concentration solution particles tend to, through collisions and electrostatic repulsion, having slightly higher diffusion speeds. This gives the effect that particle size can be underestimated in the highest particle concentration solutions. Due to a high degree of dilution prior to measurements this problem is most likely negligible in the PTA. The AuNP:BSA hydrodynamic size is within the same size range as in previous reports. Citrate capped 60 nm AuNPs in human serum albumin had a hydrodynamic diameter of 107 nm by DLS [44]. For comparison, another study shows that citrate capped 60 nm AuNPs mixed with BSA had a hydrodynamic diameter of approximately 70 nm by DLS [45]. The uncertainty related to the PTA and DLS hydrodynamic diameters can be seen in Table 1.

The sizes obtained by DLS are larger than by PTA. The difference between measured PTA and DLS sizes can be explained by the different measurement principles. With PTA the scattered light from single particles is tracked. With the DLS method, the light interferences from a collection of particles of different sizes are obtained, from which the hydrodynamic size distribution is inferred. As the size of particles increase, their relative light scattering cross section increases dramatically. This means that a small fraction of larger aggregates, barely visible by PTA analysis, can have a major impact in DLS analysis (weighting ∼Dp^6^ for small sizes). This illustrates the complementary nature of the two methods. DLS and PTA reference measurements without deposited AuNPs can be seen in Figure S1–S2 and Table S2. No differences are seen in the size of the complexes when AuNPs are deposited in BSA in PBS (150 mM salt), or in BSA in 10 mM NaPO_4_ without salt indicating that BSA form complexes with AuNPs in both conditions. The AuNP:BSA complexes formed are stable over time: DLS measurements of a sample incubated 48 h are the same as for samples incubated a few hours (Figure S3).

In addition to homocysteine and albumin, AuNPs are deposited into porcine blood serum. The AuNP concentration is calculated to 16.7±2.5 and 19.2±2.9 µg/ml for 100% and 10% serum, respectively. Blood serum is the fluid left after cells and coagulated blood components have been removed from the blood. The protein concentration is approximately 70 mg/ml in the blood serum. About half of the proteins in serum are albumin. There are also small compounds with a free thiol group, such as homocysteine, and reduced glutathione. Thus, the blood serum represents a mixture of the two complexity conditions: homocysteine and BSA. There are, however, approximately 3,700 different kinds of proteins in the serum, which creates a much more complicated situation than BSA. The AuNPs are deposited into undiluted serum and serum diluted ten times with PBS. The size of the AuNPs in the serum is determined with PTA, and DLS (Figure 5C, D and Table 1). The absorbance spectra of AuNPs in diluted serum overlap the spectra obtained in BSA, suggesting that they are the same size. In undiluted serum a red shift can be observed in the absorbance spectra towards longer wavelengths, Figure 4. This shift indicates the formation of larger AuNP:Protein complexes [44]. The size of the AuNP:Protein complexes is determined to be 75±1 nm in 10% serum by PTA. This is similar to the depositions in BSA, again suggesting that complexes of the same size are formed in the diluted serum and the BSA solution. In 100% serum, the size is determined to be 113±6 nm by PTA, suggesting that AuNPs in 100% serum form larger AuNP:Protein complexes than in 10% serum and in the BSA buffer. In 10% and 100% serums, the dominating peaks represent a DLS size of 142±8 and 198±12 nm, respectively.

The size of the AuNP:Protein complexes in 10% serum is similar to the size obtained by DLS for AuNP:BSA complexes, while deposited AuNPs form larger complexes in 100% serum. The same relation is found using the PTA. For comparison, the PTA hydrodynamic sizes determined for AuNPs in 100% and 10% serum lie in the same size range as previously reported sizes. For citrate capped AuNPs mixed with human serum, diluted 1∶1,000,000 in citrate buffer, and the hydrodynamic mode diameter by PTA is 68.5 nm. By DLS the same AuNP serum mixture shows a peak mode hydrodynamic diameter at 70.1 nm [46], the size is derived in the study from an intensity weighted distribution.

### Deposition of Agglomerate AuNPs

**Figure 6:**
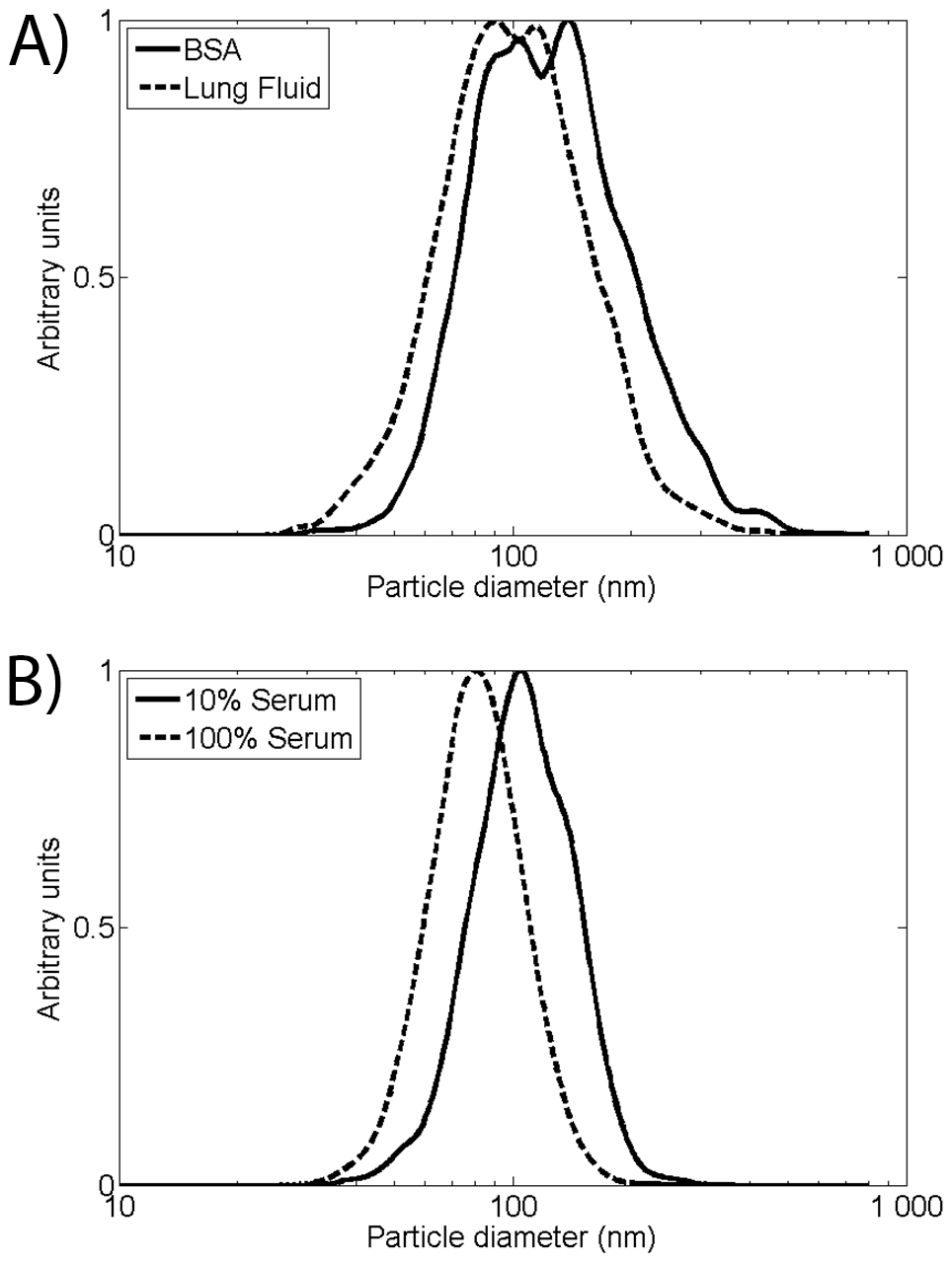
PTA characterization of AuNP agglomerates. A) PTA size measurements of AuNP:Protein complexes in BSA and lung fluid. B) PTA size measurements of AuNP:Protein complexes in 10% and 100% porcine serum.

The AuNP generation process also allows for deposition of agglomerate AuNPs (Figure 3B). As described above these particles are agglomerates formed of very small primary particles, 5–6 nm in diameter. In order to compare if differences in the surface structures affect the behavior of AuNPs in solutions, we deposited agglomerate AuNPs of 60 nm into BSA, lung fluid, and serum (Figure 6A and B). The mass concentration of AuNP agglomerates is approximately 2.0±0.3 µg/ml in BSA solution and 3.0±0.5 µg/ml in serum and lung fluid solution. In contrast to the spherical AuNP, no pink surface layer was observed after deposition of AuNP agglomerates. This can be explained by the light scattering properties of the AuNP agglomerates, governed not only by the agglomerate size but by the size of the primary particles.

In BSA a broad peak is observable ranging between 70 and 200 nm in the size distribution obtained by PTA (Figure 6A). Compared to the spherical AuNPs in BSA the distribution is significantly broader with fewer particles below 70 nm. A similar broad peak was also observed after agglomerate AuNPs was deposited into lung fluid. A general shift towards smaller hydrodynamic sizes with a narrower peak (80–100 nm) could be observed, Figure 6A.

The AuNPs deposited in 10% and 100% serum solution show distinct peak mode sizes of 81±1 nm and 107±2 nm, respectively, determined by PTA. This is similar to sizes obtained for spherical AuNPs. The deposited agglomerate AuNPs are less prone to form complexes in BSA than in serum and lung fluid. This could be explained by a low affinity between agglomerate AuNP and BSA. In serum and lung fluid there is a large variety of proteins with a varying degree of affinity for the AuNPs, this increase the probability of efficient AuNP:Protein complex formation.

No evidence is found for disintegration of the (gas phase generated) AuNP agglomerates upon deposition into the biological fluids. Inspection of the TEM images shows considerable bridging between the primary particles, thus the agglomerates can be termed hard agglomerates. This can be expected as the agglomeration predominantly takes place at elevated temperatures in the aerosol generators.

### AuNP Surface Area Compared to Protein Surface Area in Solution

The ratio of protein surface area to AuNP surface area, agglomerate and spherical, was calculated and compared in the physiological solutions. This was done in order to asses if there was sufficient homocysteine and protein to completely cover the AuNP in solution. The surface area calculated for the physiological solutions was the total cross sectional surface area of its constituents. This because the cross sectional surface area in relation to the AuNP surface area most likely determine the number of proteins or homocysteine molecules that can bind to the surface.

The cross sectional area of a homocysteine molecule is in this study approximated by calculating the average van der Waals radius for the molecule [47]. The cross sectional surface area, based on the approximate van der Waals radius and not taking atomic overlap into account, is then 4.3·10^−15^ cm^2^ per molecule. The total number of homocysteine molecules in the solution, 5 mM, is 3.0·10^18^/ml. The minimum number of homocysteine to cover the Relating this to the total deposited AuNP surface area, 10.3±1.3 cm^2^/ml, is the minimum number concentration to cover the particles with homocysteine is on average 2.4·10^15^/#/ml (5.2·10^4^ cm^2^/ml). The difference in the number concentration required to completely cover the deposited AuNP with homocysteine surface area than nanoparticle surface area in the solution and the actual concentration spans 3 orders of magnitude.

The total cross sectional protein surface area in a BSA solution of 35 mg/ml is 1.4·10^5^ cm^2^/ml. This is based on a cross sectional surface area of 4.6·10^−17^ m^2^/protein [43]. For the experiments with spherical AuNP the total deposited surface area is 0.7±0.1 cm^2^/ml, for the experiments with agglomerate AuNP the deposited surface area was 0.9±0.1 cm^2^/ml. For the AuNP agglomerates a specific surface area of 45 m^2^/g was used for calculations [48].

The total cross sectional protein surface area of the porcine serum solution is due to the large number of proteins of different size and structure impossible to calculate. For the lung fluid solution no calculation is performed due to the complexity of the solution. A useful approximation for the serum solutions can be made from the albumin concentration. In 10% serum there is 3.5 mg/ml albumin corresponding to approximately 1.4·10^4^ cm^2^/ml cross sectional protein surface area. For 100% serum the cross sectional surface area is approximated by surface area concentration of the BSA solution, 1.4·10^5^ cm^2^/ml.

This approximation most likely represents a minimum total cross sectional surface area for the serum solutions. This is due to the serum protein concentration is twofold that of albumin and the greater variety of proteins in serum. The total deposited spherical AuNP surface area in the 100% serum solution and 10% serum solution was 1.0±0.1 cm^2^/ml and 1.2±0.2 cm^2^/ml respectively. The total deposited agglomerate surface area was 1.4±0.2 cm^2^/ml.

In conclusion there is sufficient homocysteine/protein surface area to completely cover all AuNP deposited in the physiological solutions.

### Deposition in Porcine Lung Fluid

**Figure 7:**
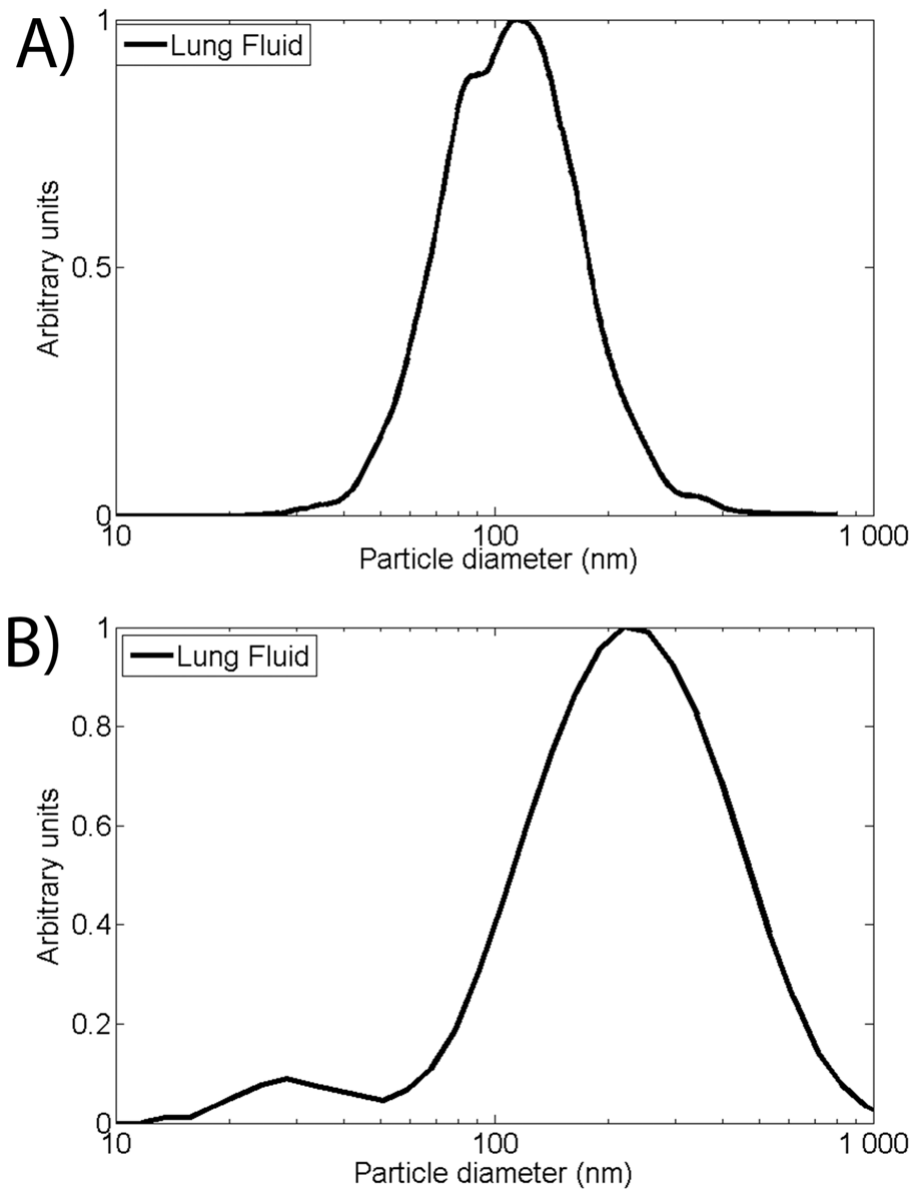
PTA and DLS characterization of AuNP:lung fluid complexes. A) PTA size measurement of AuNP:lung fluid complexes. B) DLS size measurements of AuNP:lung fluid complexes.

From a physiological perspective the most relevant exposure route for nanoparticles is deposition in the respiratory tract. Thus, AuNPs are deposited into porcine lung fluid in the same fashion as for the other physiological fluids. The lung fluid was collected by rinsing a porcine lung with PBS. Typical constituents of lung fluid are organic surfactants and proteins. The deposited mass of spherical AuNP corresponds to a concentration of 16.7±2.5 µg/ml. The hydrodynamic mode diameter found is 110±7 and 265±17 nm determined by PTA and DLS respectively (Table 1 and Figure 7A and B).

For this study no accurate approximation of the total cross sectional surface area is performed. The total deposited spherical AuNP surface area was 1.0±0.2 cm^2^/ml. The total deposited agglomerate surface area was 1.4±0.2 cm^2^/ml.

### Corona Proteins Bound to Gold Particles Deposited in Porcine Blood Serum

**Figure 8:**
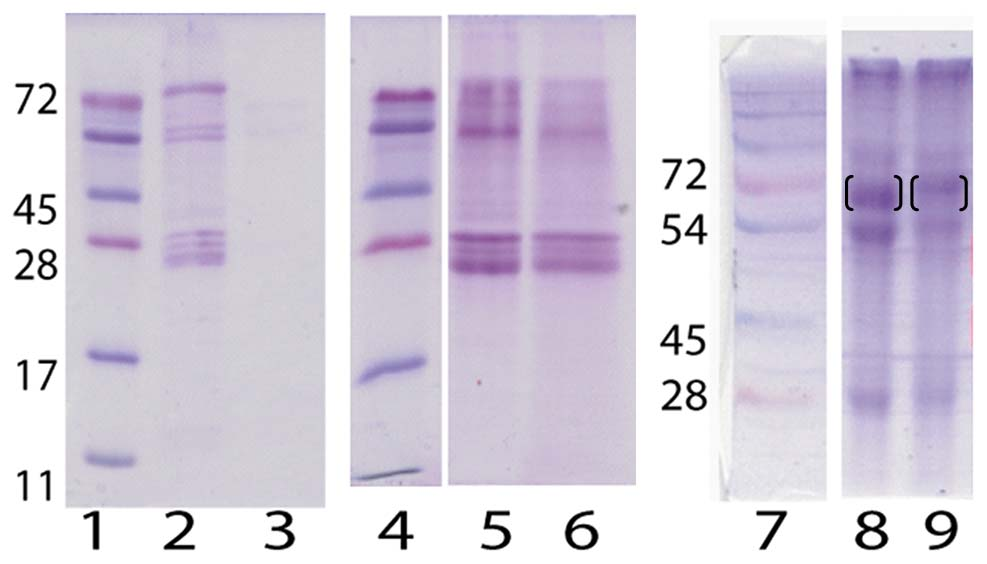
Protein corona characterizations of AuNP in 10% serum solution. Lanes 1 and 4: Molecular weight (kDa) of known proteins on a 15% SDS-PAGE. Lane 2: Serum proteins bound to spherical AuNPs. Lane 3: Serum proteins found in the pellet without deposited AuNP. Lane 5: Serum proteins bound to spherical AuNPs. Lane 6: Serum proteins bound to agglomerate AuNPs. Lane 7. Molecular weight (kDa) of known proteins on a 12% SDS-PAGE. Lanes 8 and 9: Proteins on spherical AuNPs deposited into BSA solution and transferred/incubated into 10% porcine serum before centrifugation and corona characterization (68 kDa band in brackets).

The rationale behind deposition of AuNPs into biological fluids is that proteins and other biomolecules will bind to and thereby form AuNP:Protein complexes in solution. To visualize the protein binding in 100% and 10% serum, the AuNP:Protein complexes are separated from unbound proteins by centrifugation. The resulting pellets are washed repeatedly with buffer; bound proteins are desorbed by detergent (SDS, sodium dodecyl sulphate) and separated by size by SDS-PAGE (polyacryl gel electrophoresis). This favors the identification of proteins bound with high affinity to the AuNPs. Proteins with low affinity are removed together with unbound proteins in the washing procedure. No pellets are recovered in 100% serum, and no visualization of protein binding by electrophoresis could be performed. However, in 10% serum a clear pellet is recovered after centrifugation. There is a distinct set of proteins bound to the AuNPs (Figure 8: Lanes 2, 5, and 6).

Interestingly, this protein pattern differ from the protein pattern found on citrate capped gold particles of similar size in fetal calf serum [15], [49], suggesting that the deposited AuNPs bind different proteins. On the SDS-PAGE, albumin migrates with a molecular weight of about 68 kDa, there are no visible or only barely visible protein bands in that area for Lanes 2, 5–6. Indicating that in competition with other serum proteins, albumin does not bind to AuNPs. Lanes 5 and 6 in Figure 8 compare the proteins bound to spherical and agglomerate AuNPs. No significant differences can be seen between the two types of structures with regards to protein binding.

To test the stability of the AuNP:BSA complexes in competition with other proteins, the complexes are transferred into 10% serum solution. The transfer is performed by separating the complexes by centrifugation, resulting in the formation of pellet. The supernatant is removed and 10% serum solution added, after which the pellet is dissolved by vortexing of the solution. After one hour incubation in 10% porcine serum, the AuNP:Protein complexes are centrifuged and the bound proteins separated on a SDS-PAGE (Figure 8: Lanes 8 and 9). The protein pattern is similar as to when the AuNPs were deposited directly into diluted serum. Note that proteins migrate different distances on PAGES with different acrylamide concentrations. However, there is an extra protein band with a molecular weight around 68 kDa (Figure 8 lane 8–9), which most likely correspond to albumin, suggesting that some albumin is left on the AuNPs after one hour incubation in porcine serum.

A comparison of the size of the AuNP:Protein complexes in different solutions reveals an increasing size in increasing complexity of proteins (Table 1). The smallest AuNP:Protein complexes are found in the BSA buffer solution, larger or similar sizes are found in 10% serum and the largest sizes in 100% serum and lung fluid. The larger structures in 100% serum and lung fluid can be explained by a greater variety of proteins and a higher concentration of larger proteins. Larger proteins would give rise to a thicker corona. The differences in the AuNP:Protein complexes in 10% and 100% serum is also manifested in the centrifugation experiments. In the 10% serum, the AuNP:Protein complexes are easily centrifuged but not in the 100% serum, indicating complexes with less density. Many nanoparticles, including AuNPs have been reported to bind lipoproteins in blood serum or plasma [50]. Lipoproteins are complexes between apolipoproteins and phospholipids, triglycerides, and cholesterol. They have low density, 0.95–1.06 mg/ml, and a diameter of around 10–1000 nm. Thus, the binding of lipoproteins can explain both why the size of the AuNPs is larger in 100% and why they are more difficult to pellet.

## Summary

In conclusion, as airborne particles move into physiological solution both their morphological characteristics and the characteristics of the solution play a key role in the subsequent Particle:protein complex formation. Knowing the characteristics of these complexes will be vital to fully understand the particles toxicity and biological fate.

AuNP:Protein complexes of distinct sizes are formed and are characterized using an array of methods. Upon deposition in protein solutions, such as BSA, lung fluid and serum AuNP:Protein complexes form. The corona composition is also different from that of AuNPs generated in liquid suspension. If deposited into a solution without proteins, with only a single biomolecule such as homocysteine, larger micron sized aggregates form as flakes. The morphological properties of the deposited particles have also been shown to alter its characteristics in solution.

The combination of methodology in this study represents an important step forward in air-liquid interface research. It is possible to generate airborne particles of known and variable characteristics and deposit them into physiological solutions of increasing complexity. In these solutions, the generated particles form complexes with the ambient proteins and biomolecules and form new biological entities. It is possible to characterize these complexes by a complementary measurement array of DLS, PTA and UV-Vis spectroscopy to determine their state and stability in solution, and compare with the state while airborne, characterized by mobility, mass and morphology.

A natural next step would be to employ this complete method in a specially designed air-liquid interface chamber [29], simulating the conditions in the lung. In such a chamber, it is possible to expose cells to airborne particles, and deposit a known fraction onto the cells. The chamber conditions the aerosol to physiological conditions during the deposition process. By this process it would be possible to study the change in characteristics of the nanoparticles as they pass into the natural physiological solution produced by epithelial cells while studying adverse effects on the cells.

## Materials and Methods

### Generation and Characterization of Gold Nanoparticles in Gas Phase

An aerosol of AuNPs was generated using high temperature evaporation and condensation [31]. This was followed by a sintering furnace for the generation of spherical particles. A Bipolar charger followed by a Differential Mobility Analyzer was used to select 60 nm particles. The size distribution of the produced particles was also determined with the DMA connected to an electrometer, forming a Scanning Mobility Particle Sizer (SMPS). For deposition, the airborne AuNPs were led into an electrostatic precipitator (ESP). A complete outline of the system can be seen in Figure S4 and is described in detail in elsewhere [48], [51]. In generating the aerosol particles, the temperature of the evaporation-condensation furnace was kept at 1800°C. The temperature of the sintering furnace, where particle agglomerates were sintered into spheres, was kept at 600°C. For generating an aerosol of agglomerate AuNP with a peak mode diameter of 60 nm, the generation temperature had to be slightly decreased. In order to produce 60 nm spherical particles, the aerosol was sintered and then passed through the DMA to select particles of 60 nm. For 60 nm agglomerates, the sintering furnace was inactive while the DMA was set, as for the spheres, to select 60 nm particles. The uncertainty in the selected particle size by the DMA is less than 10%. The repeatability is however significantly higher.

#### DMA principle of operation

The DMA consists of two concentric cylinders, where the particles are introduced near the outer cylinder and transported through the DMA by a sheath flow [32]. If no additional force is applied, the particles introduced at the outer cylinder will not be selected. However, by applying a voltage between the two cylinders, part of the charged particles will be forced towards the inner cylinder. Particles of a certain electrical mobility will be selected at an exit slit, illustrated in Figure S5. In this way, a monodisperse particle distribution is generated after the DMA. The carrier gas flow rate through the system was 1.7 l/min and the DMA sheath flow rate was 10 l/min. The DMA in the system was of Vienna [52] type with a length of 11 cm, an inner radius of 25 mm and an outer radius of 33 mm. The electrical mobility, *Z*, of the particles is described in the DMA as:
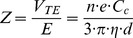



The terminal velocity, *V_TE_*, in the electrical field *E* is related to the number of elementary charges, *n*, multiplied by *e*, divided by the viscosity, *η*, and equivalent mobility particle diameter *d*. The DMA-transfer function (broadening due to the instrument) of the 60 nm particles is illustrated in Figure 1 here and in Figure S5A, B and C. The particles are, before entering the DMA, charged using a bipolar charger, generating a Boltzmann-like particle charge distribution [53]. Hence, a fraction of particles will be doubly charged. Doubly charged particles selected at the same DMA voltage have the same electrical mobility as the singly charged particles but a larger size. As a consequence, a small fraction of the selected particles will have a larger mobility diameter. In this study it was 85.5 nm (Table S1). For the particle size distribution generated and at the DMA settings used in this experiment, only 3–6% of the particles selected are doubly charged, and thus for practical purposes nearly negligible.

In combination with an electrometer, and a bipolar charger the DMA forms what is referred to as a Scanning Mobility Particle Spectrometer (SMPS). The mobility and particle number concentration data are combined in a data inversion, creating a time resolved number size distribution.

#### APM principle

The APM consists of two rotating cylinders. By balancing the centrifugal force against an electrostatic force, only one particle mass can pass through the volume at one time between the rotating cylinders [33]. The relationship between mobility diameter and particle (aggregate) mass can be determined by combining a DMA with an APM [48], [54].

#### TEM characterization

For further characterization of the particles generated, agglomerate and sintered (spherical) particles were deposited on a lacey carbon-coated Cu transmission electron microscopy (TEM) grid. A 300 kV field emission TEM (JEOL 3000 F), equipped with an X-ray energy dispersive spectrometer (XEDS) was used to analyze the morphology and crystal structure of the generated AuNPs, and to confirm their chemical composition.

### Deposition

The ESP consists of a metal plate where a voltage of 6 kV is applied during deposition [38]. A glass beaker with a diameter of 5 cm containing a known amount of physiological fluid is placed on the metal electrode plate. Since the DMA used operates with a positive voltage (selecting negatively charged particles), only negatively charged particles are fed to the ESP. When the positive voltage of the ESP is applied to the metal plate, the particles are thus forced down and deposited into the biological fluid.

Using the average total number concentration during each of the deposition events the total deposited AuNP number could be calculated. For the applied ESP voltage 100% deposition efficiency is expected. The total calculated AuNP number was then divided by the total collected physiological solution volume, after which mass/nr/surface area concentrations could be calculated. The AuNPs were deposited into 5 buffer solutions of physiological fluids: pure PBS buffer solution, 5 mM homocysteine (Sigma) in PBS, 45 mg/ml BSA (Sigma) in PBS, 100/10% porcine blood serum diluted with PBS, and porcine lung fluid.

The porcine lung fluid was extracted by rinsing porcine lungs repeatedly with PBS. The lungs were massaged during the rinsing procedure to ensure the PBS to reach the lower parts of the lungs. The solution is centrifuged to remove blood constituents and other impurities before usage. The animals were part of another acute study at Lund University and were sedated and ventilated during the procedure. Since they were part of another study, of acute effect, no specific ethical permit was required for the extraction of lung fluid and blood serum in accordance with European and Swedish legislation.

### Characterization in Solution

In solution the particles were analyzed using dynamic light scattering (Malvern Nano S equipped with a red 632.8 nm HeNe laser). Scattered light was detected at 175°. The homocysteine solution with AuNPs was prepared as a dilution series of 100, 10, 1 and 0.1% AuNP concentrations. The dilute was a homocysteine buffer. The BSA solution was also prepared as a dilution series; the dilute was a BSA solution in a PBS buffer. Dilution series were prepared as 100, 10 and 1% AuNP concentrations. 100% serum, 10% serum, and lung fluid were measured without preparing dilution steps. For all physiological fluids, with or without AuNPs, the distribution analysis result was used for size determination in solution. The distribution analysis refers to a multiple exponential fit to the correlation function, allowing multiple size modes to be calculated. For the homocysteine no distribution analysis was possible so a Z-average value was determined. A number conversion was attempted. Since albumin and other proteins/biomolecules are dominant with regards to number, it proved difficult to discern any other peaks than that of albumin. However, a tendency for a number size peak could be observed around 40–100 nm in serum and BSA. The hydrodynamic diameter determined and referred to by DLS is the peak, or so-called mode, diameter for the intensity weighted size distribution.

Samples were also measured using PTA, (model NanoSight LM10 HS) equipped with a 405 nm laser. All samples were diluted in milliQ H_2_O to suitable concentration for measurement. The dilutions used were: 100000× for 100% serum samples, 10000× for 10% serum samples, 100× for BSA samples, and 100× for lung fluid. Processing parameters: blur = 9×9, Min Expected Particle Size = 100 nm, and Min Track = Auto. The hydrodynamic diameter determined and referred to by PTA is the peak, or so-called mode diameter for the number size distribution obtained by fitting a lognormal function to the determined size distributions. For each DLS and PTA record, the mode diameter for the AuNP:Protein complex was identified and averaged with a standard deviation (Table 1). In addition, the distributions were averaged together into a single figure (Figure 4). The total number of records for each solution was 10 for DLS. For PTA, the number of records was 5 for lung fluid, BSA and 10% Serum, and 3 for 100% serum.

UV absorbance measurements were performed using a Kanomax spectrometer. All measurements were done in Quartz cuvette. Background signals were obtained from the same biological fluids as depositions were done into and subtracted.

The particle protein corona was analyzed for the AuNPs in the physiological fluids using SDS-PAGE. The samples, 1 ml, were centrifuged 15 minutes at 13,000 rpm, after which the supernatant was removed and 0.5 ml PBS added. The pellet was dispersed and moved into new tubes to minimize unspecific protein binding on the tube walls. This procedure was repeated twice. After the last centrifugation, the supernatant was removed and a 10 µl SDS-PAGE loading buffer was added to the samples in order to dissolve proteins bound to the particles. The proteins were separated by SDS-PAGE and visualized by coomassie blue.

### Reference Measurements

The PTA analysis was performed in 100,000 and 10,000 times dilution with milliQ water for 100% serum and 10% serum AuNP solution, respectively. This means that the protein concentration after dilution is the same in 10% and 100% serum, but that the AuNP concentration is ten times lower in 100% serum.

The reference measurements of 100% and 10% serum dilutions without AuNPs are as expected virtually identical, since the protein concentration is the same. However, the solutions with deposited AuNPs differ from their corresponding reference measurements. By PTA there are more particles per ml in the 10% serum AuNP solution than in the 100% serum AuNP solution. Both 100% and 10% serum reference solutions have the same protein concentration due to the dilution prior to PTA measurements, and the same size signature when measured by PTA. The ratio between the number of deposited AuNPs in the 10% serum and 100% serum solution should be 10 to 1. As can be seen in Figure S6, this is not the case in the two solutions with AuNPs. In conclusion, it is not possible to deduct background particles from a PTA measurement with particles, using a reference measurement without particles. This is most likely due to the different scattering properties between biomolecules and AuNPs.

### Ethics Statement

The porcine lungs and blood plasma was obtained from another study being conducted at Lund University, Swedish Governmental Ethics Committee (permit nr:172-11). The study investigated acute cardiac effects on sedated and ventilated pigs. Lung fluid and serum was recovered from the lungs and blood of the deceased pigs. Both studies follow Swedish and European legislation with regards to ethical approval, The Council of the European Communities Directive EG 2010/63/EU, The Swedish Animal Welfare Act 1988∶534, The Swedish Animal Welfare Ordinance 1988∶539, The Swedish provision regarding the use of animals for scientific purposes SJVFS 2012∶26 L150.

## Supporting Information

Figure S1 PTA reference measurements of the physiological solutions without deposited AuNP.
(TIF)

Figure S2 DLS reference measurements of the physiological solutions without deposited AuNP.
(TIF)

Figure S3 DLS measurement of spherical AuNP:BSA complexes hours after and 24 hours after deposition.
(TIF)

Figure S4 Outline of the experimental system for AuNP characterization in aerosol phase and deposition into solution.
(TIF)

Figure S5 Principal of differential mobility analyzer operation. A) One electrical mobility is suitable to pass through the differential mobility analyzers electrical field. Smaller or larger sizes do not exit the instrument. B) The electrical mobility is altered if the particles carry more than one electrical charge, positive or negative. Larger particles gain increased mobility and pass as smaller particles, as is the case with the 85 nm AuNP in the study. C) Due to a geometrical broadening in the differential mobility analyzer, evident from studying (A), both smaller and larger particles are also selected for a given mobility size. The distribution is called the instruments transfer function. The geometric standard deviation of the selected particles is <1.1.
(TIF)

Figure S6 100% and 10% serum reference measurements with their corresponding AuNP:Serum measurements.
(TIF)

Table S1 Total spherical AuNP particle number, mass and surface area concentrations deposited into each of the physiological buffers and the percentage of 85.5 nm deposited double charged particles.
(DOCX)

Table S2 Peak mode sizes for reference solutions by DLS and PTA. Bold text indicate the dominating mode.
(DOCX)
