# Correction: Direct Deposition of Gas Phase Generated Aerosol Gold Nanoparticles into Biological Fluids - Corona Formation and Particle Size Shifts

**DOI:** 10.1371/annotation/63c7e7ce-df36-482a-991b-a05a4e477a82

**Published:** 2013-10-15

**Authors:** Christian R. Svensson, Maria E. Messing, Martin Lundqvist, Alexander Schollin, Knut Deppert, Joakim H. Pagels, Jenny Rissler, Tommy Cedervall

A funding organization and grant were incorrectly omitted from the Funding Statement. The following sentence should be included in the Funding Statement: We thank the EC for co-funds, NanoReg (FP7-310584).

