# Supplementary figures and images for: Direct Deposition of Gas Phase Generated Aerosol Gold Nanoparticles into Biological Fluids - Corona Formation and Particle Size Shifts
Source: PLoS One. 2013 Sep 27;8(9):e74702. doi: 10.1371/journal.pone.0074702 (PMC3785473; doi:10.1371/journal.pone.0074702)

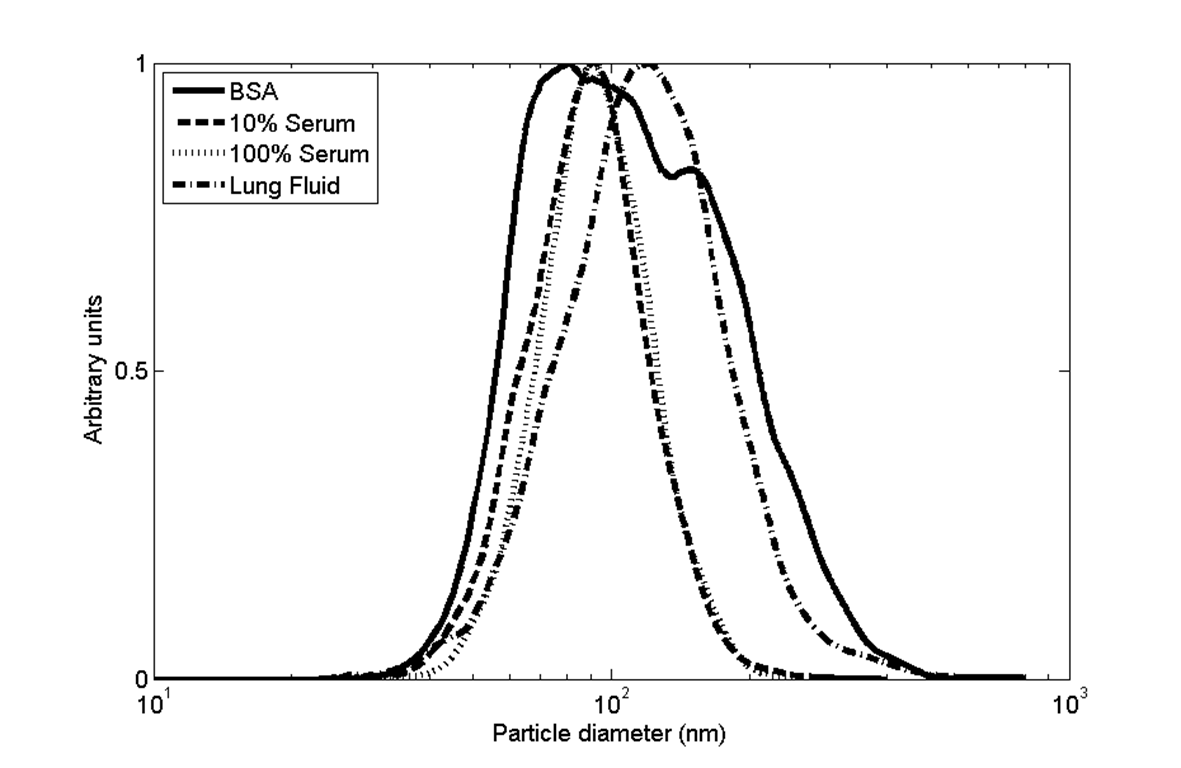

Supplement: Figure S1 — PTA reference measurements of the physiological solutions without deposited AuNP. (TIF) [file pone.0074702.s001.tif]

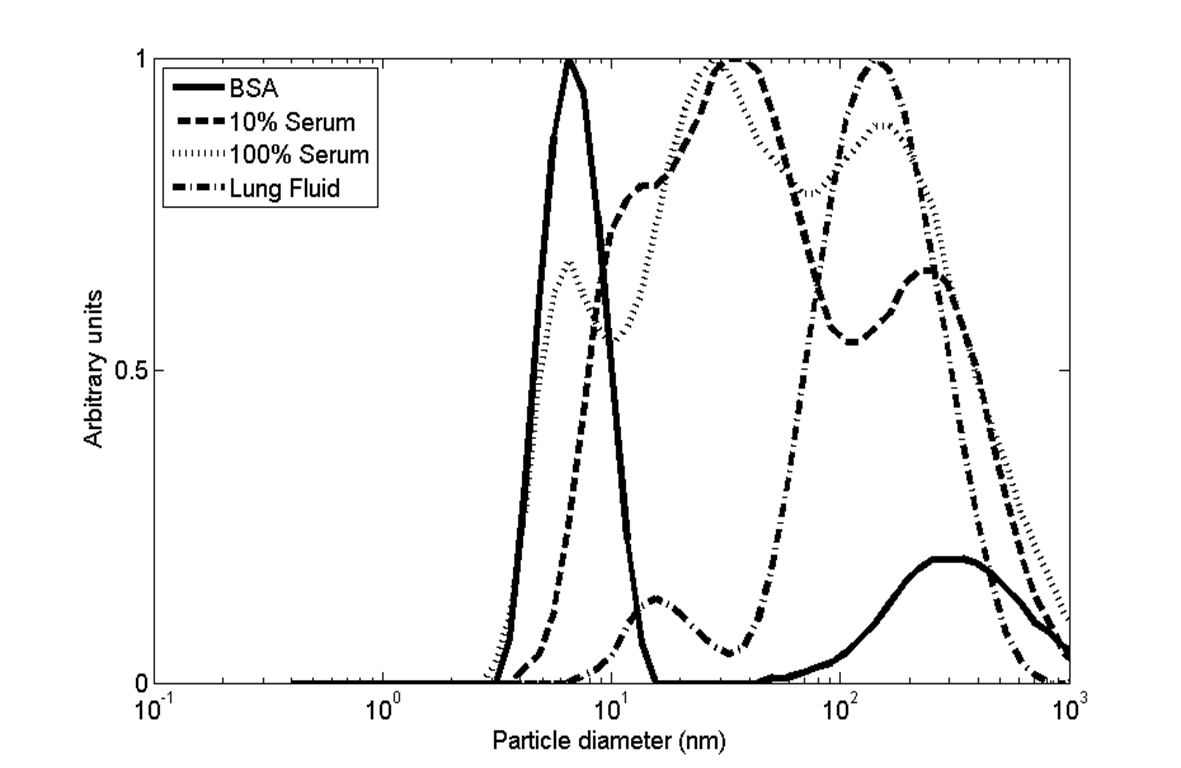

Supplement: Figure S2 — DLS reference measurements of the physiological solutions without deposited AuNP. (TIF) [file pone.0074702.s002.tif]

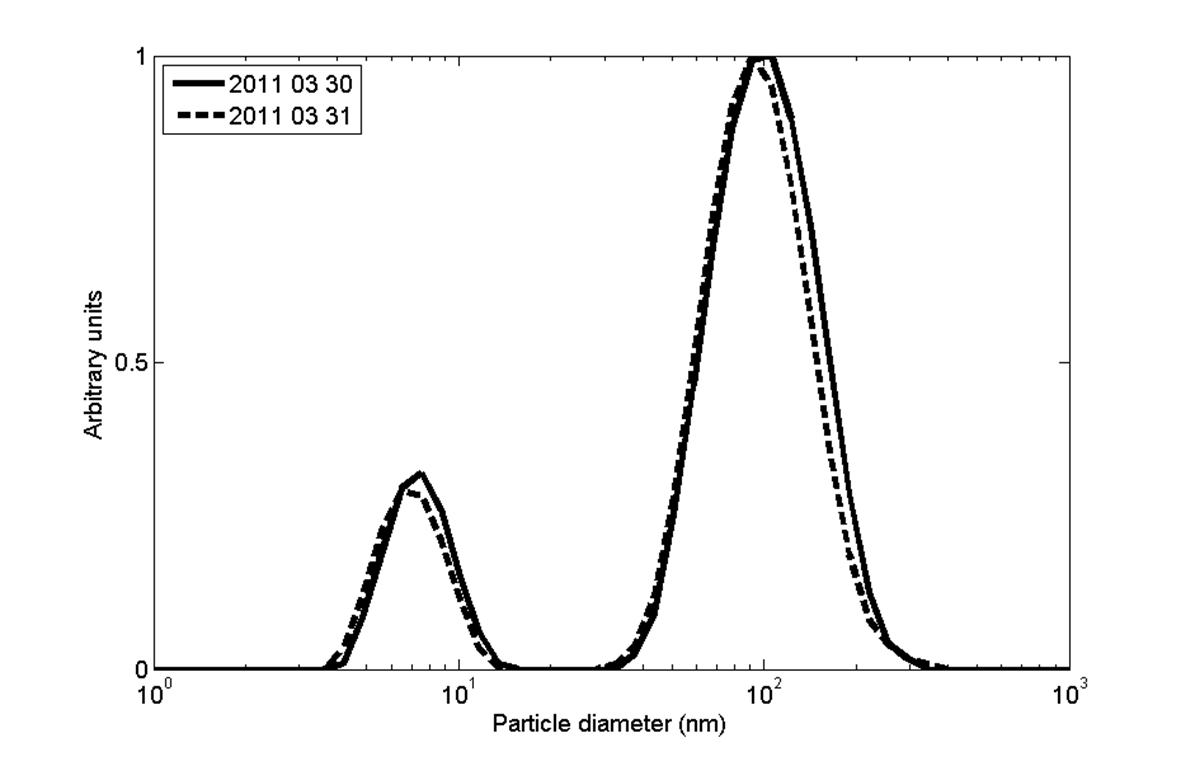

Supplement: Figure S3 — DLS measurement of spherical AuNP:BSA complexes hours after and 24 hours after deposition. (TIF) [file pone.0074702.s003.tif]

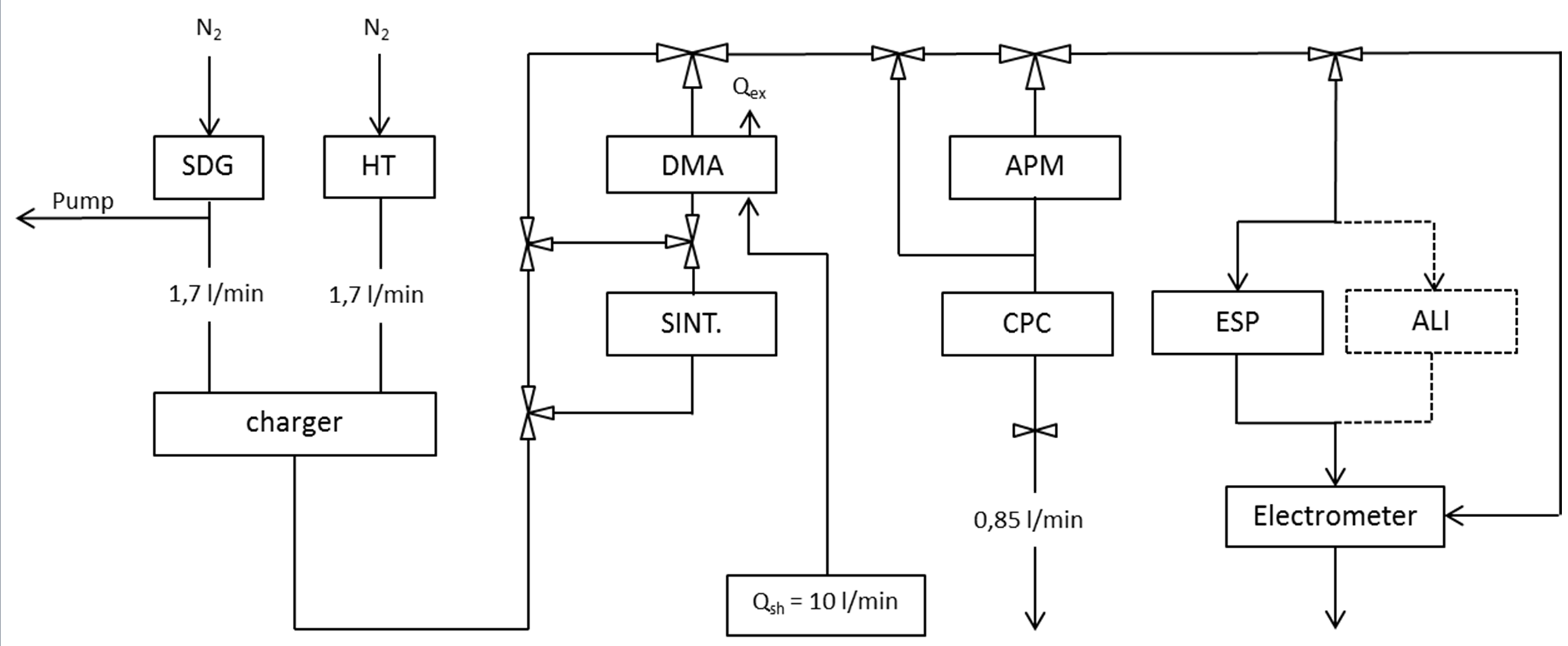

Supplement: Figure S4 — Outline of the experimental system for AuNP characterization in aerosol phase and deposition into solution. (TIF) [file pone.0074702.s004.tif]

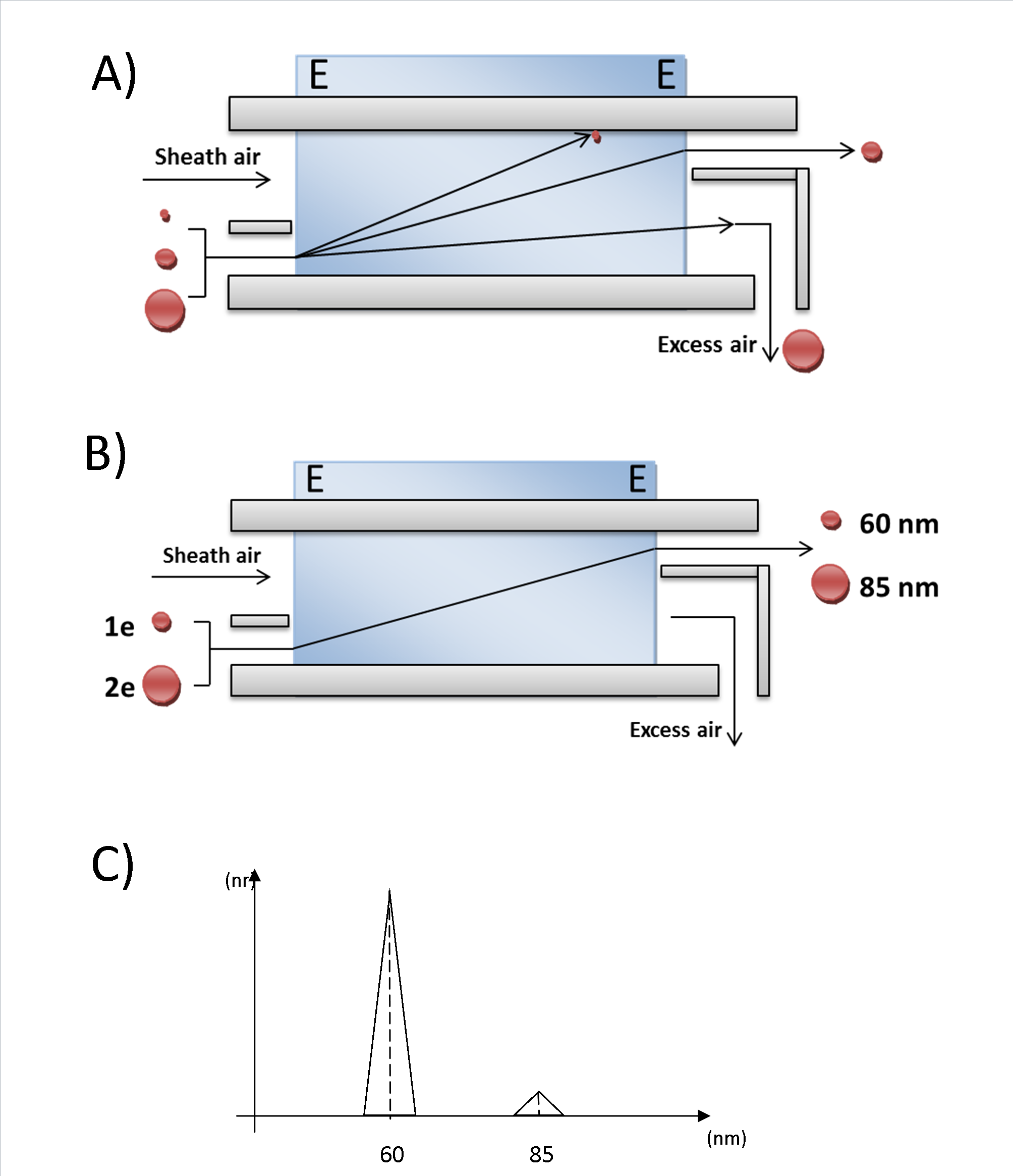

Supplement: Figure S5 — Principal of differential mobility analyzer operation. A) One electrical mobility is suitable to pass through the differential mobility analyzers electrical field. Smaller or larger sizes do not exit the instrument. B) The electrical mobility is altered if the particles carry more than one electrical charge, positive or negative. Larger particles gain increased mobility and pass as smaller particles, as is the case with the 85 nm AuNP in the study. C) Due to a geometrical broadening in the differential mobility analyzer, evident from studying (A), both smaller and larger particles are also selected for a given mobility size. The distribution is called the instruments transfer function. The geometric standard deviation of the selected particles is <1.1. (TIF) [file pone.0074702.s005.tif]

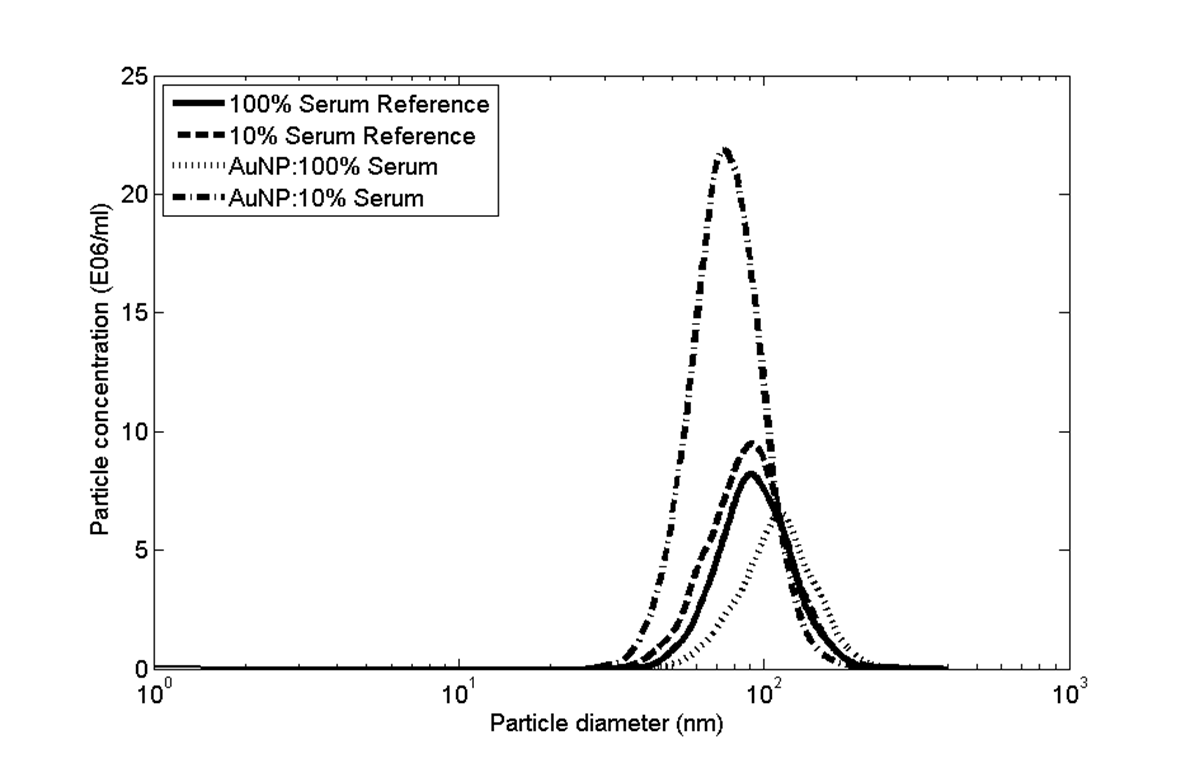

Supplement: Figure S6 — 100% and 10% serum reference measurements with their corresponding AuNP:Serum measurements. (TIF) [file pone.0074702.s006.tif]
