# Supplementary material for: Direct Deposition of Gas Phase Generated Aerosol Gold Nanoparticles into Biological Fluids - Corona Formation and Particle Size Shifts
Source: PLoS One. 2013 Sep 27;8(9):e74702. doi: 10.1371/journal.pone.0074702 (PMC3785473; doi:10.1371/journal.pone.0074702)
Supplement: Table S1 — Total spherical AuNP particle number, mass and surface area concentrations deposited into each of the physiological buffers and the percentage of 85.5 nm deposited double charged particles. (DOCX) [file pone.0074702.s007.docx]

|  | Total Particle # / ml (±std) | % Double charged 85.5 nm of particle number | Total particle mass μg/ml (±std) | Total AuNP cm2 / ml (±std) |
| --- | --- | --- | --- | --- |
| Homocysteine | 9.15 · 10^10^ ± 1.19 · 10^10^ | 3.1 | 168.3 ± 22.0 | 10.3 ± 1.3 |
| BSA | 6.38 · 10^9^ ± 2.85 · 10^8^ | 4.1 | 11.7 ± 0.5 | 0.7 ± 0.1 |
| 100% Serum | 9.06 · 10^9^ ± 1.37 · 10^9^ | 6.5 | 16.7 ± 2.5 | 1.0 ± 0.1 |
| 10% Serum | 1.04 · 10^10^ ± 1.58 · 10^9^ | 6.5 | 19.2 ± 2.9 | 1.2 ± 0.2 |
| Lung Fluid | 9.07 · 10^9^ ± 1.37 · 10^9^ | 6.5 | 16.7 ± 2.5 | 1.03 ± 0.2 |
