# Supplementary material for: Direct Deposition of Gas Phase Generated Aerosol Gold Nanoparticles into Biological Fluids - Corona Formation and Particle Size Shifts
Source: PLoS One. 2013 Sep 27;8(9):e74702. doi: 10.1371/journal.pone.0074702 (PMC3785473; doi:10.1371/journal.pone.0074702)
Supplement: Table S2 — Peak mode sizes for reference solutions by DLS and PTA. Bold text indicate the dominating mode. (DOCX) [file pone.0074702.s008.docx]

|  | DLS (nm, std) | PTA (nm, std) |
| --- | --- | --- |
| Homocysteine reference | 170.3 (47.8) | - |
| BSA reference | **7.13** (0.07) / 393.4 (152.4) | 104 (29.7) |
| 100 % Serum reference | **34.6** (16.5) / 282.8 (176.9) / 8.05 (2.01) | 92.0 (3.27) |
| 10 % Serum reference | **43.5** (11.4) / 12.2 (1.73) / 294.9 (62.3) | 91.8 (4.07) |
| Lung fluid reference | **171.7** (7.37) / 17.7 (4.64) | 114.0 (9.40) |
